# Involvement of the multidisciplinary team and outcomes in inpatient rehabilitation among patients with inflammatory rheumatic disease

**DOI:** 10.1186/s12891-016-0870-9

**Published:** 2016-01-13

**Authors:** Till Uhlig, Olav Bjørneboe, Frode Krøll, Øyvind Palm, Inge Christoffer Olsen, Margreth Grotle

**Affiliations:** Department of Rheumatology, From the National Advisory Unit on Rehabilitation in Rheumatology, Diakonhjemmet Hospital, Oslo, Norway; Institute of Clinical Medicine, University of Oslo, Oslo, Norway; Martina Hansens Hospital, Bærum, Norway; Rheumatism Hospital, Lillehammmer, Norway; Østfold Hospital, Sarpsborg, Norway; Oslo and Akershus University College of Applied Sciences, Oslo, Norway; Oslo University Hospital, FORMI, Oslo, Norway

**Keywords:** Patient care team, Rehabilitation, Outcome assessment, Rheumatoid arthritis, Psoriatic arthritis, Ankylosing spondylitis

## Abstract

**Background:**

The last decades have for patients with inflammatory rheumatic diseases seen a shift towards more physically active rehabilitation programs, often provided as out-patients with less use of inpatient facilities. There is little research on which effect the multidisciplinary team has on health outcomes for patients with rheumatoid arthritis, psoriatic arthritis, ankylosing spondylitis, and connective tissue disease. This study examined patient reported outcomes for patients with inflammatory rheumatic diseases receiving rehabilitation care as inpatients in departments of rheumatology, and studied how number of consultations with the multidisciplinary team affected these clinical outcomes.

**Methods:**

Patients with inflammatory rheumatic diseases were included in a multi-center prospective observational study if rehabilitation was considered a focus during an inpatient stay at four departments of rheumatology. At admission, discharge, and after 3 and 6 months, 317 patients were assessed with patients reported outcomes (PRO) including health assessment questionnaire (HAQ), short-form 36 (SF-36), pain, fatigue, patient global assessment of disease activity, self-efficacy scales, rheumatoid arthritis disease activity index (RADAI), and SF-6D utility. Patients stated consultations with the multidisciplinary team.

**Results:**

Improvements were short-lived, and at 6 months follow-up period only mental health, pain and utility remained improved with small effect sizes. Extensive involvement of health professionals was not associated with improved outcomes.

**Conclusions:**

Patients with inflammatory rheumatic disease receiving inpatient multidisciplinary rehabilitation had small and mainly short-term improvements in most PROs. High use of the multidisciplinary team did not enhance or preserve rehabilitation outcomes in inflammatory rheumatic conditions when admitted as inpatients.

## Background

Musculoskeletal diseases are among the most regular complaints in the general population [1]. Recommmendatations highlight a multidisciplinary team care approach for the best management in musculoskeletal conditions [2], but there is limited evidence for effects. Effective management of RA requires a range of nonpharmacological interventions [3], which are delivered by members of the multidisciplinary team. Applicable approaches are for example a combination of physical exercise and cognitive behavioural therapy [4] or a combination of physical exercise, surgery and diet [5], and often consist of broad education programmes [6–8]. The multidisciplinary team may consist of rheumatologists, rehabilitation specialists, occupational therapists, physical therapists, social workers, nurses, manual therapists, podiatrists, dieticians, psychologists, vocational counsellors and orthopaedic surgeons.

Inpatient multidisciplinary care over a short time has demonstrated effect in active patients with rheumatoid arthritis (RA) [9] and ankylosing spondylitis [10] as well as in patients with inflammatory and non-inflammatory musculoskeletal diseases [11]. A systematic review found inpatient multidisciplinary care more effective compared to regular outpatient care [12], while some studies found equivalent clinical effects between inpatient and day-care [13, 14] or outpatient [15] team care programmes.

Care in the field of rheumatology has in the last decades moved towards more physically active rehabilitation programs provided as out-patients, a development which could provide access to a larger multidisciplinary team. Another development during recent years has been that patients referred to multidisciplinary rehabilitation present with lower levels of physical disability than previously, but still benefit from multidisciplinary rehabilitation for their inflammatory joint diseases in an era where effective pharmacological therapies are widely available [16]. Thus, given the effectiveness of inpatient rehabilitation, we hypothesized that rehabilitation also is effective when provided in rheumatology departments, and also that the extent to which the multidisciplinary team is involved would be reflected in rehabilitation outcomes among patients with specific inflammatory rheumatic diseases.

The objective of this study was thus to examine 6-month outcomes of patients with different inflammatory rheumatic diseases who were admitted to departments of rheumatology with a need for multidisciplinary rehabilitation. We especially examined whether the extent of using the multidisciplinary team of health care professionals (HCP) or of single health professions, before or during rehabilitation, was related to levels and changes in health outcomes.

## Methods

### Design

The study was designed as a multi-center, longitudinal observational study, and relevant patients at the participating departments of rheumatology were consecutively recruited and followed during the rehabilitation stay, at discharge, and after 6 months. Four departments of rheumatology in Eastern Norway participated over a 24-month period.

### Inclusion criteria

Patients were eligible if they had existing inflammatory rheumatic disease at the time of admission, and hospitalization was anticipated to last for at least one week. Specifically, when consecutive patients were admitted as inpatients for any reason to departments of rheumatology, then during the medical consultation at admission a possible need for rehabilitation was assessed. Such a need for rehabilitation during the stay was defined as planned involvement of at least two HCP (in addition to rheumatologist and nurse), thus fulfilling a case requirement for delivering multidisciplinary care. Thus, rehabilitation could be the major or a minor focus of the inpatient stay. Patients were at age 18 years and higher and signed informed consent to participate in the study. The study was approved by the regional ethics committee for Eastern Norway (REK 2004-13499).

### Measurements

The diagnosis was recorded based on referral and examination at admission. Once included, patients completed questionnaires on socio-demographic variables: age, gender, level of education, marital status, work status, height and weight. At admission patients indicated which HCP they had consulted during the last year (general practitioner, rheumatologist, orthopedic surgeon, nurse at general practitioner’s office, nurse at the rheumatologist office, physiotherapist, occupational therapist, manual therapist, social worker, and psychologist).

Self-reported patient reported outcomes (PROs) included pain, fatigue, and patient global assessment of disease severity on 100 mm visual analog scales (VAS). Physical disability was assessed by the Health Assessment Questionnaire disability index (HAQ, 0-3) [17] with upgrading of scores due to devices or help from another person. Physical function and mental function were also assessed using the Short-Form 36 (SF-36) Health Survey with physical (PCS) and mental (MCS) component summaries on 0-100 scales (100 = best functioning) [18]. The Arthritis Self-Efficacy Scales [19] for pain and symptoms with range 10-100 (100 = best) were used to assess self-efficacy or the believe in the capability to carry out a behaviour, and which reflects a concept of perceived control. Rheumatoid arthritis disease activity index (RADAI) has questions on disease activity, joint tenderness, pain, morning stiffness and perceived joint pain in 16 joint areas [20]. The utility measure (SF-6D) was derived from the responses to the SF-36 questionnaire based on an algorithm developed by Brazier [21] and can be used for analyses in health economy (range 0.3-1, 1 = perfect health).

At discharge from the hospital, and at three and six months follow-up, patients again completed questionnaires with identical PROs as during admission. At discharge patients again ticked off from a list consultations during the stay with ten members of the multidisciplinary team (rheumatologist, orthopaedic surgeon, nurse, physiotherapist, occupational therapist, social worker, psychologist, dietician, pharmacist, orthopaedic engineer).

### Statistical analysis

Descriptive statistics are presented as means with standard deviation (SD) or 95 % confidence intervals for continuous data, or as percentages for counts. Effect sizes for change of health outcomes at follow-up time points were assessed as standardized response means (SRM), the mean change divided by standard deviation at baseline. The effect sizes were interpreted as small (SRM 0.2-0.5), moderate (SRM 0.5-0.8), and large (SRM >0.8) [22].

Consultation with HCP during rehabilitation was grouped into tertiles: high HCP use (>6 professions), medium HCP use (5 professions), and low HCP use (<4 professions). For the year preceding inpatient rehabilitation the tertiles were >5, 3-4, and <2 professions, respectively.

Comparisons between completers and non-completers of the study, and between high and low HCP use were performed with independent samples t-tests for continuous and chi square tests for categorical variables. Analysis of variance (ANOVA) and chi square tests compared baseline values for the different diagnoses. PRO values at discharge, and at 3- and 6 months follow-up were compared with baseline using paired samples t-tests.

Linear regressions mixed models with random intercept by patient were applied including data from all patients over the whole study period, defining individual health outcomes as dependent variables, and analysing time, age, gender, diagnosis, education, marital status, use of HCP and baseline outcome value as independent variables. We then included the interaction between use of multidisciplinary team*time in the regression model, thus analysing the time dependent effects of high versus low HCP use on specified outcomes.

*P*-values <0.05 were considered significant. No correction for multiple testing was performed. SPSS version 21.0 (IBM) was used for the statistical analyses.

## Results

The demographic and clinical baseline characteristics of all 373 included patients and those 317 (85.0 %) completing the 6 months follow-up are displayed in Table 1. Completers were older, more often female, married or cohabiting, and had a shorter hospital stay than non-completers. There were no statistically significant differences in baseline PROs between completers and non-completers.

**Table 1:** Demographic and baseline clinical characteristics for all patients

	All (*n* = 373)	Completers (*n* = 317)	Non-completers (*n* = 56)	*P*-value^a^
Age (years)	52.6 (14.2)	53.3 (14.0)	49.0 (15.0)	0.034
Gender (% females)	74	77	59	0.004
Married/cohabiting (%)	64.6	68.7	48.2	0.045
Education (College/University, %)	27.2	25.6	37.2	0.14
Rheumatoid arthritis (%)	52	53	48	0.14
Disease duration (yrs)	13.4 (11.0)	13.1 (10.9)	14.7 (11.0)	0.32
Hospital days	14.1 (6.9)	13.4 (6.5)	17.9 (8.0)	0.001
Working (%)	35.3	34.3	41.5	0.31
HAQ (0-3)	1.23 (0.67)	1.21 (0.66)	1.33 (1.71)	0.20
SF-36 PCS (0-100)	30.3 (10.1)	30.1 (10.0)	30.2 (10.8)	0.95
SF-36 MCS (0-100)	44.9 (12.7)	45.4 (12.4)	42.0 (14.2)	0.07
Pain (0-100)	49.9 (24.7)	49.9 (24.7)	50.5 (25.2)	0.85
Fatigue (0-100)	59.9 (27.4)	59.2 (27.5)	63.8 (26.5)	0.26
Patient global (0-100)	48.8 (25.4)	48.3 (24.8)	52.2 (28.5)	0.29
RADAI (0-10)	4.5 (2.0)	4.4 (2.1)	4.5 (2.0)	0.86

Values are means with standard deviation or %

^a^ Completers vs. non-completers

*HAQ* Health Assessment Questionnaire, *SF-36* Short Form 36, *PCS* Physical component summary, *MCS* mental component summary, *SES* Self-Efficacy Scales, *RADAI* Rheumatoid Arthritis Disease Activity Index

RA was the most prevalent diagnosis in among included patients, observed in 196 patients (52.5 %), followed by ankylosing spondylitis in 72 (19.3 %) and psoriatic arthritis in 66 (17.7 %) patients, while 39 patients (10.5 %) had other inflammatory rheumatic diagnoses (systemic lupus erythematosus, systemic sclerosis, mixed connective tissue disease, palindromic rheumatism, vasculitis). Baseline characteristics in these four groups were similar for most variables, but some statistically significant differences were seen: age and physical disability (HAQ) were significantly higher for RA, disease duration and length of hospital stay shortest for psoriatic arthritis, while the frequency of females was lowest for AS, and the level of fatigue highest in the group with other inflammatory diagnoses (data not shown, ANOVA *p* < 0.05).

### Changes in PRO

Table 2 display results for unadjusted PROs at admission, discharge, 3 and 6 months follow-up for study completers. At discharge all the PROs were statistically significantly improved, but the effects wore off, and at 6 months follow-up only pain, fatigue, SF-6D, SF-36 MCS and patient global assessment of disease activity were statistically significantly improved as compared to baseline assessment at admission to the department, but corresponded to no more than at most small effect sizes at 3- and 6 months follow-up.

**Table 2:** Patient reported outcomes for completers at admission and follow-up (*n* = 317)

	Admission/Baseline	Departure	3 months	6 months
HAQ (0-3)	1.21 (0.66)	1.16 (0.67)**	1.20 (0.66)	1.22 (0.68)
SF-36 PCS (0-100)	30.3 (10.0)	31.4 (9.6)**	31.0 (9.9)	31.1 (9.6)
SF-36 MCS (0-100)	45.4 (12.4)	46.9 (11.5)*	47.3 (11.8)**	47.3 (11.8)**
Pain (0-100)	49.8 (24.7)	40.6 (23.6)**	44.0 (24.2)**	45.7 (24.6)**
Fatigue (0-100)	59.2 (27.5)	53.8 (28.3)**	53.3 (28.0)**	54.5 (27.5)**
Patient global (0-100)	48.3 (24.8)	39.6 (22.8)**	44.5 (24.7)*	45.4 (24.4)*
SES pain (10-100)	54.0 (16.9)	57.5 (16.8)**	54.4 (16.7)	54.5 (17.1)
SES symptoms (10-100)	61.0 (15.2)	64.9 (15.3)**	62.8 (15.6)*	61.2 (15.8)
RADAI (0-10)	4.5 (2.1)	3.9 (1.9)**	4.3 (2.1)	4.4 (2.1)
Utility (SF-6D, 0-1)	0.596 (0.11)	0.618 (0.11)**	0.622 (0.12)**	0.621 (0.12)**

Values are means (standard deviation)

*p < 0.05 **p < 0.01 (paired t-test) versus baseline

*HAQ* Health Assessment Questionnaire, *SF-36* Short Form 36, *PCS* Physical component summary, *MCS* mental component summary, *SES* Self-Efficacy Scales, *RADAI* Rheumatoid Arthritis Disease Activity Index

**Fig. 1:**
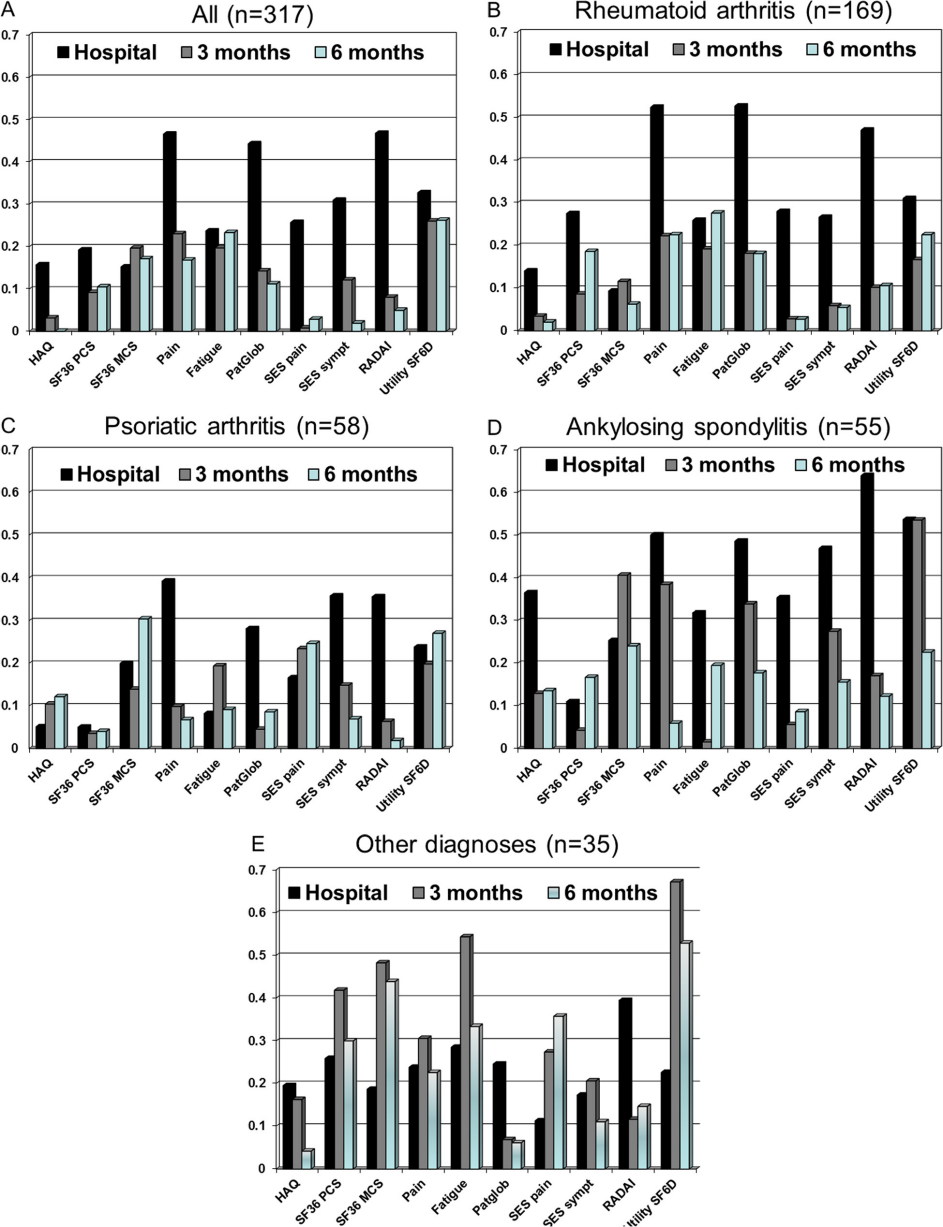
Change scores (standardized response means) for rehabilitation outcomes during hospital stay, and after 3 and 6 months in (**a**) all patients, and patients with (**b**) rheumatoid arthritis, (**c**) psoriatic arthritis, (**d**) ankylosing spondylitis, (**e**) other diagnoses. *HAQ* Health Assessment Questionnaire, *SF36 PCS* Short Form 36 physical component summary, *SF36 MCS* Short Form 36 mental component summary; *SF-6D* Short Form 6 dimensions, *RADAI* Rheumatoid Arthritis Disease Activity Index, *Patglob* Patient Global Assessment of disease activity, *SES* = Self-Efficacy Scales

The standardized improvements of PRO are shown in Fig. 1a-e with means of SRM at departure, 3 month and 6 month follow-up. SRMs are shown for all patients and according to the specific inflammatory rheumatic diagnosis. While up to moderate effect sizes were seen at departure, at 3- and 6-months follow-up only, only small effect sizes around 0.2 were observed. The effect sizes were highest for pain, fatigue and SF6D-utility.

Changes outcomes were then adjusted for a number of covariates and for baseline values and are shown in Table 3, providing estimated marginal means with 95 % CI. For easier interpretation, statistically significant findings are given in bold types. Of all PRO only SF-36 MCS, pain, and SF-6D utility were still significantly improved at 6 months follow-up and fatigue was statistically significantly improved until 3 months follow-up.

**Table 3:** Changes from baseline scores for patient reported outcomes (PRO)

	Change (*n* = 317)		
	Discharge - Baseline	3 months – Baseline	6 months - Baseline
HAQ	**-0.09 (-0.13 to -0.2)**	-0.04 (-0.14 to 0.03)	0.03 (-0.09 to 0.03)
SF-36 PCS	**1.1 (0.1 to 2.1)**	0.4 (-0.6 to 1.4)	0.6 (-0.5 to 1.6)
SF36 MCS	**2.1 (0.6 to 3.5)**	**2.3 (0.9 to 3.8)**	**2.3 (0.8 to 3.8)**
Pain	**-9.4 (-12.5 to -6.4)**	**-5.0 (-8.1 to -1.9)**	**-3.8 (-7.0 to -0.7)**
Fatigue	**-5.1 (-8.3 to -1.8)**	**-3.5 (-6.9 to -0.2)**	-3.0 (-6.3 to 0.4)
Patient global	**-8.9 (-12.1 to -5.7)**	-2.5 (-5.7 to 0.7)	-2.0 (-5.3 to 1.2)
SES pain	**3.28 (1.1 to 5.5)**	-0.1 (-2.4 to 2.1)	-0.1 (-2.4 to 2.2)
SES symptoms	**4.1 (2.1 to 6.0)**	1.8 (-0.1 to 3.8)	-0.1 (-2.1 to 1.9)
RADAI	**-0.66 (-0.90 to -0.41)**	-0.11 (-0.37 to 0.14)	-0.07 (-0.33 to 0.18)
SF6D	**0.028 (0.014 to 0.042)**	**0.027 (0.012 to 0.041)**	**0.028 (0.014 to 0.042)**

Values are estimated marginal means with 95 % confidence intervals, adjusted for age, gender, diagnosis, education, marital status, use of health professionals and baseline

Statistically significant change scores shown in bold types

*HAQ* Health Assessment Questionnaire, *SF-36* Short Form 36, *PCS* Physical component summary, *MCS* mental component summary, *SES* Self-Efficacy Scales, *RADAI* Rheumatoid Arthritis Disease Activity Index

### Consultation with HCP

**Fig. 2:**
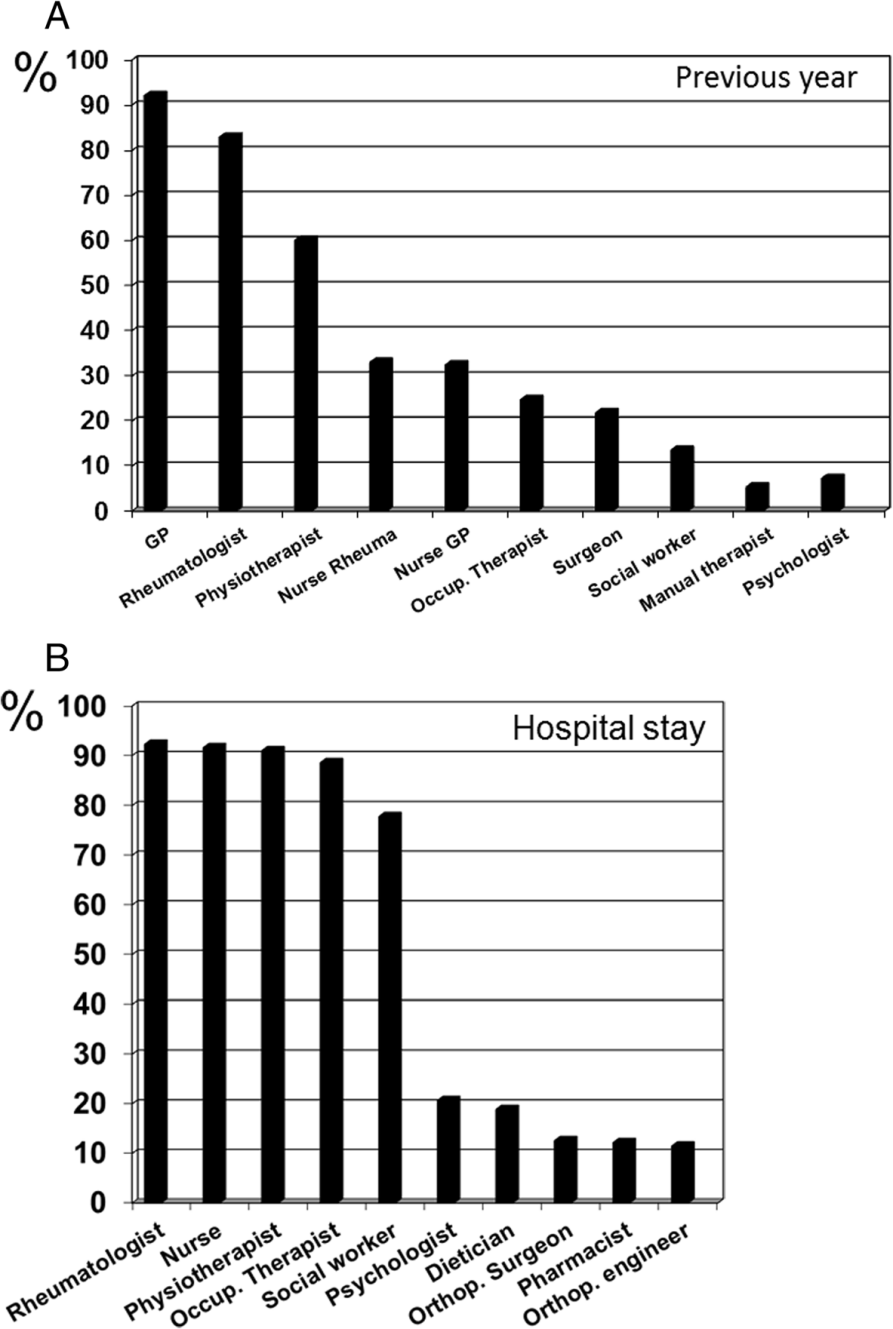
Consultations of patients (*n* = 317) with health care professions (**a**) in the year preceding (**b**) during the hospital stay. *GP* General Practitioner, *Occup. Therapist* Occupational Therapist, *Orthop. Surgeon* Orthopedic Surgeon, *Orthop. Engineer* Orthopedic Engineer

During the year preceding the admission for the rehabilitation stay patients had median 3 (interquartile range 2-5) HCP consultations, with a range 0 to 9 HCP. The corresponding numbers for HCP consultation during inpatient rehabilitation was median 5 (interquartile range 4-6) consultations, ranging from 0 to 9. The frequency of consultations with the individual HCP in the year prior to the study and during the hospital stay is shown in Fig. 2a-b.

We did not find any consistent association between consultations with any specific single HCP prior to or during rehabilitation, and change in health outcomes at discharge or follow-up (data not shown).

PRO levels were in general not statistically significantly different between high and low use of HCP (highest vs. lowest tertile) in the year before or during the rehabilitation stay (Table 4), but for physical function (HAQ and SF-36 PCS), fatigue and utility at admission levels were statistically significantly worse for patients with high use of HCP in the year before admission.

**Table 4:** Baseline PROs for low and high use^a^ of HCP the year before and during hospital stay

Variable	Last year before rehabilitation (*n* = 317)			During rehabilitation stay (*n* = 317)		
	Low use (<2) (*n* = 94)	High use (>5) (*n* = 93)	*p*- value	Low use (<4) (*n* = 85)	High use (>6) (*n* = 84)	*P*-value
HAQ	1.08 (0.65)	1.42 (0.66)	<0.001	1.20 (0.68)	1.35 (0.68)	0.60
SF36 PCS	32.4 (9.7)	27.6 (10.1)	0.001	30.2 (9.8)	27.7 (10.4)	0.11
SF36 MCS	44.7 (12.1)	45.2 (13.0)	0.79	44.7 (11.7)	43 (13.4)	0.40
Pain	52.3 (25.4)	53.7 (25.4)	0.71	50.1 (26.8)	51.9 (24.0)	0.66
Fatigue	56.2 (28.3)	65.2 (25.2)	0.023	58.9 (26.7)	64.8 (25.6)	0.15
Patient global	49.2 (27.2)	52.6 (23.0)	0.38	50.0 (26.0)	50.5 (23.7)	0.90
SES pain	55.6 (16.2)	54.2 (16.8)	0.57	52.9 (18.8)	53.3 (14.3)	0.90
SES symptoms	60.2 (14.7)	62.6 (13.9)	0.28	59.9 (14.4)	61.2 (14.6)	0.57
RADAI	4.40 (2.08)	4.80 (2.07)	0.20	4.72 (2.26)	4.60 (1.93)	0.72
SF6D	0.61 (0.12)	0.57 (0.10)	0.038	0.60 (0.11)	0.55 (0.10)	0.002

^a^ Use of health care professionals is grouped in lowest and highest tertile. The intermediate tertile for last year before rehabilitation included 126 patients (missing *n* = 4), and the tertile for during the rehabilitation stay included 134 patients (missing *n* = 14)

Values are means with standard variation and *p*-value from independent samples t-test

*PRO* Patient reported outcomes, *HCP* Health Care professionals, *HAQ* Health Assessment Questionnaire, *SF-36* Short Form 36, *PCS* Physical component summary, *MCS* mental component summary, *SES* Self-Efficacy Scales, *RADAI* Rheumatoid Arthritis Disease Activity Index

Table 5 presents differences in patient reported outcomes between the high and the low HCP-use groups. These differences are given for these groups comparing their respective changes over defined follow-up periods, adjusting for covariates. The difference was calculated by subtracting the change score in the low HCP-use group from that in high HCP-use” group (Table 5). Findings showed that no major differences in change scores between high and low HCP users were seen through all PROs and for all follow-up periods. However, the numerical differences indicated less improvement in the high HCP use group, i.e. no further reduction compared to the low HCP-use group. On the contrary, in the high HCP-use group some statistically significant differences with less improvement were seen at 3-months follow-up (RADAI, SF-36 physical component, and patient global). For example, while all patients after three months had a numerical improvement in patient global assessment of disease activity (-2.5 mm) (Table 3), there was a deterioration of +6.6 mm in the high HCP-use group as compared to the low HCP-use (Table 5).

**Table 5:** Differences (∆) between high and low users of the multidisciplinary team observed for change in patient reported outcomes during follow-up periods

Outcome	High versus low use of health professionals		
	Discharge	3 months	6 months
∆HAQ	0.09, *p* = 0.10	0.08, *p* = 0.13	0.03, *p* = 0.57
∆SF-36 PCS	0.3, *p* = 0.77	**-2.4,** ***p*** **= 0.023**	-0.2, *p* = 0.85
∆SF36 MCS	-0.71, *p* = 0.62	0.60, *p* = 0.68	0.59, *p* = 0.69
∆Fatigue	1.3, *p* = 0.69	2.3, *p* = 0.49	0.2, *p* = 0.95
∆Pain	2.2, *p* = 0.48	5.9, *p* = 0.06	3.0, *p* = 0.34
∆Patient global	0.8, *p* = 0.79	**6.6,** ***p*** **= 0.04**	0.7, *p* = 0.85
∆SES pain	2.5, *p* = 0.26	0.4, *p* = 0.87	4.2, *p* = 0.07
∆SES symptoms	-0.2, *p* = 0.91	-1.5, *p* = 0.44	0.6, *p* = 0.74
∆RADAI	0.33, *p* = 0.17	**0.62,** ***p*** **= 0.012**	0.17, *p* = 0.31
∆SF6D	-0.011, *p* = 0.44	-0.015, *p* = 0.29	-0.005, *p* = 0.74

Values are estimated marginal means with *p*-values, adjusted for age, gender, diagnosis, education, marital status, use of health professionals and baseline values

Statistically significant differences in change scores are shown in bold types

*HAQ* Health Assessment Questionnaire, *SF-36* Short Form 36, *PCS* Physical component summary, *MCS* mental component summary, *SES* Self-Efficacy Scales, *RADAI* Rheumatoid Arthritis Disease Activity Index

## Discussion

In this study we examined the effects of consultations with HCP on clinical outcomes in patients with inflammatory rheumatic diseases admitted to departments of rheumatology. For all patient groups, independent of diagnosis and extent of HCP use, improvements for PRO were mainly temporary and had largely levelled off already at 3 months of the 6 months follow-up after the rehabilitation stay. More importantly, involving a high number of HCP or any specific HCP was not associated with better health, measured as any of the PROs, neither immediately after the rehabilitation stay nor delayed during the next 3-6 months. Changes in health outcomes were generally small. Almost all numeric and all statistically significant findings pointed towards better baseline function and larger improvements during the stay in the group with less involvement of HCP.

These findings are counterintuitive, as all efforts by HCP are directed to alleviating disease burden and enhance coping with the disease. Most likely unmeasured, and maybe unmeasurable factors contribute to involving HPC in patients with perceived problems. The patients will then not necessarily demonstrate better PRO in follow-up assessments. Thus, our findings contribute to a hypothesis that high use of the multidisciplinary team is a marker of disability and of specific rehabilitation needs, indicating more complicated disease where one may not expect major improvement of health status. We find it important to clearly state this conundrum, so health care workers who spend much time with highly disabled patients with inflammatory rheumatic disease will not go discouraged when their rehabilitation efforts do not show as improvements demonstrated by means of standardized PROs.

Future research mandates to quantify study elements of efforts made by the individual HCP. Only a randomized controlled trial which compares high and low levels of multidisciplinary rehabilitation, could study whether the broad multidisciplinary team provides better health outcomes. This should also include proper goal setting process and coordinated multidisciplinary team-care in many of these patients, which is now recommended [23].

Rehabilitation in inflammatory joint diseases has also in other studies shown at least short lived improvements. A three-week multidisciplinary day-care program has early shown to be beneficial in patients with RA [24], and a number of studies has shown at least temporary improvement after multidisciplinary team care in mixed populations [25, 26], especially in patients with rheumatoid arthritis [13, 14, 16, 24, 27–29] and ankylosing spondylitis [10]. As a typical finding scores in patients with inflammatory rheumatic disease after 6 months again have reached baseline [30], suggesting supportive follow-up interventions to possibly maintain outcome improvement [31]. Disappointing in the results of our study was also the observed lack of lasting improvement of self-efficacy, which could be expected to improve after involvement of the multidisciplinary team. Our findings indicate that self-management issues were not effectively addressed even in patients with high use of HCP in departments of rheumatology.

Multidisciplinary team care has developed during the last years with increasing focus on evidence-based evaluation [32]. Due to the questionable responsiveness of multidisciplinary team care [15], evaluation should be function specific and overall physical disability [33]. We therefore applied a wide range of outcome measures, including physical function, pain, fatigue, health related quality of life, but also self-efficacy and a utility measure which could be applied for health economy. An improvement in SF-36 derived utility of about 0.025 would – if maintained for one year - correspond to a gain of 2.5 quality adjusted life years in 100 patients.

While our study did not examine the specific content and intensity of rehabilitation, it investigated the involvement of HCP, constituting the multidisciplinary team. Strengths of this study are the relative large sample size from four departments of rheumatology, inclusion of a typical spectre of inflammatory rheumatic diseases, and broad outcome assessments which also included disease activity, self-efficacy and utility.

A number of limitations apply to our study. First, included patients reported on use of HCP, but not further on the contents of these consultations or rehabilitation interventions. Further, rehabilitation in our study was most likely given individualised to patients’ needs parallel to other treatment modalities for the rheumatic disease at regular hospital departments, which is again part of multidisciplinary treatment in itself, i.e. we did not evaluate pharmaceutical modalities. Also, more tailored rehabilitation interventions could have led to greater outcome effects. We gave patients in the questionnaires the opportunity to state events which during the rehabilitation stay could have affected outcomes, e.g. initiation of biological medication, surgical procedures, or infections. Only a minority of patients made such statements, and we did not systematically register all other medication, for example analgetics, preventing us from specifically accounting for these factors in our analyses. Another limitation is the observational design of the study which prevents causal conclusions. Finally, our results are restricted to patients with inflammatory arthropathies, where some need for rehabilitation was seen during regular admissions at departments of rheumatology. This clinical setting does represent predefined major rehabilitation needs, and our findings can thus not be generalized to rehabilitation effects.

More focus on goals and other aspects of rehabilitation could have led to better health outcomes than seen in our study. In fact, we know that the different departments of rheumatology do not have a unified practice of rehabilitation, and we do not know which specific rehabilitation modalities were applied in our patients.

## Conclusions

This study addressed rehabilitation outcomes until 6 months in inpatients with inflammatory rheumatic diseases, finding small and mainly short-term improvements in a wide range of PRO. High use as compared to low use of HCP in a multidisciplinary team did not enhance or preserve rehabilitation outcomes. High use of HCP was, however, associated to poorer PRO at baseline, and these findings illustrate that in clinical practice resources are used according to clinical need. Research using randomized controlled trials is warranted to study how effective multidisciplinary teams are in the rehabilitation of inflammatory rheumatic diseases.

## Abbreviations

ANOVA: analysis of variance
HAQ: health assessment questionnaire
HCP: health care professionals
MCS: mental component summary
PRO: patient reported outcome
PCS: physical component summary
RA: rheumatoid arthritis
RADAI: rheumatoid arthritis disease activity index
SF-36: short-form 36 health survey
SD: standard deviation
SRM: standardized response mean
VAS: visual analogue scale
